# Advance care planning discussions – Perspectives from oncology patients with advanced-stage disease

**DOI:** 10.1017/S1478951525000069

**Published:** 2025-04-07

**Authors:** Delinda Pendleton, Brian L. Egleston, Carolyn Y. Fang

**Affiliations:** 1 Patient Experience and Strategic Clinical Initiatives, Fox Chase Cancer Center, Philadelphia, PA, USA; 2 Department of Biostatistics and Bioinformatics, Fox Chase Cancer Center, Philadelphia, PA, USA; 3 Cancer Prevention and Control Program, Fox Chase Cancer Center, Philadelphia, PA, USA

**Keywords:** Advance care planning, advance care planning discussions, patient perspectives

## Abstract

Evidence in the literature confirms that advance care planning (ACP) discussions, assumed to benefit patients, their caregivers, and clinicians, occur at various rates, lower than intended and not as early as thought to be beneficial. The literature, however, provides limited reasons why this trend continues. When ACP discussions do occur, particularly between clinicians and patients with advanced-stage cancer, we have even less understanding of the ACP experience. Thus, the goal of our study was to characterize patient beliefs and experiences pertaining to ACP discussions, and to explore factors that may be associated with patient experiences. One hundred adults with advanced cancer participated in this cross-sectional survey study. The majority (63%) had heard about ACP, and 81% believed that health care providers should have ACP discussions with their patients. However, only 23% of participants in this sample had an ACP discussion. Among those who reported having an ACP discussion, 74% indicated that they spoke with family members about ACP, and 48% reported having spoken with their oncologist about ACP. Among those who had not had an ACP discussion with a health care provider, commonly reported reasons for not doing so included the respondent’s desire to speak with family members first and the perception that one was not sick enough to need such a discussion yet. These findings can be used to inform the development of future interventions to improve provider communication about ACP and enhance patient experiences around ACP discussions.

## Introduction

It is estimated that 13% to 25% of all Medicare spending goes toward care for people during their last year of life (Riley and Lubitz 2010), including a large portion during the last six months of life (Duncan et al. 2019). Patients with cancer malignancies contribute significantly to the burden of resources utilized in emergency departments (Rivera et al. 2017) and during hospital admissions (Kobo et al. 2021). According to several studies, variations exist in the quality and costs of end-of-life care, preferences, and palliative outcomes for cancer patients (Davis et al. 2023b; Duncan et al. 2019; French and Kelly 2016; Gomes et al. 2010). In addition, “the health care provided to patients with advanced-stage cancer does not always correlate with what is known about their preferences for care” (Khan et al. 2014). This mismatch may be due, in part, to inadequate or the absence of conversations regarding advance care planning (ACP). The literature suggests that these conversations are limited and occur at various rates (Abdul-Razzak et al. 2016; Bekker et al. 2022; Houben et al. 2014; Palmer et al. 2021). In addition, the current literature does not offer significant patient and family perspectives regarding their opinions about ACP or experiences with these discussions. Thus, the goal of the present study was to characterize patient beliefs and experiences pertaining to ACP discussions in the context of advanced cancer. These data will identify opportunities for improvement related to ACP discussions with oncology patients and highlight strategies for enhancing the ACP process in the oncology context.

## Materials and methods

### Participants and procedures

This study was approved by the Fox Chase Cancer Center (FCCC) Institutional Review Board (IRB). After receiving IRB approval, potential participants were identified through the FCCC tumor registry data base. To be eligible, patients must have (1) been diagnosed with advanced-stage cancer; (2) be at least six months from date of diagnosis; and (3) received treatment at FCCC within the calendar years of 2018–2021. The patient’s primary oncologist was contacted to obtain approval in approaching the patient prior to distribution of the questionnaire. Patients who were cleared to be approached were contacted (by phone or in-person in the clinic) by a research assistant and invited to participate in the study. The research assistant provided an overview of the study purpose and obtained written informed consent from all study participants prior to beginning any study activities.

### Measures

The survey was distributed in paper form. The items included in this survey were drawn from existing surveys that assess patient perspectives regarding ACP discussions and included the following measures.

*Assessment of ACP Experience*. Six items assessing patients’ prior experiences with ACP were drawn from the California Healthcare Foundation’s study (California Health care Foundation-CHCF-EOL 2012). Participants were asked about their experiences with ACP, including (1) whether or not they had heard about ACPs; (2) how much they have thought about ACPs; (3) how important it was to have discussions about ACPs; (4) how comfortable they were personally with having an ACP discussion; and (5) if they think health care providers should have ACP discussions with their patients. At the end of this assessment, participants were asked if they ever had an ACP discussion with a doctor or other healthcare provider.

Those participants who reported having had an ACP discussion were directed to complete 4 additional items drawn from the Advance Care Planning Team Process Improvement Project Patient Survey (Advance Care Planning Collaborative Research and Innovations Opportunities-ACP CRIO 2017) and the Behaviors in ACP and Actions Survey (Kassam et al. 2017). These items queried participants regarding with whom they spoke about ACP and the topics discussed, selecting from a multiple choice list of options for each.

*Barriers to ACP*. Participants who reported that they had not had an ACP discussion were directed to answer 3 items assessing their (1) reasons for not having an ACP discussion with their doctor or health care provider; (2) desire to have an ACP discussion; and (3) level of comfort in having an ACP discussion with their doctor or other health care provider, mental health professional, spiritual advisor, partner, parents, children, other family members, or close friends. These items were drawn from the Kaiser Health Tracking Poll (Clark et al. 2018) and the Massachusetts Statewide Population-Based Survey (DiJulio et al. 2015), which have been utilized in prior studies.

*Participant Characteristics*. In the last section of the survey, participants completed items pertaining to their demographic characteristics, including sex, age, race/ethnicity, education level, and marital status. Information about the participant’s clinical status, including cancer site and ECOG functional status, was abstracted from the electronic medical record (EMR) by study staff. In addition, whether an Advance Directive had been received by the clinical team and noted in the EMR (yes/no) was recorded by study staff.

### Analytic plan

Means, standard deviations, proportions were used to characterize the sample. We used T-tests, Wilcoxon rank-sum tests, and Fisher’s exact tests to compare differences between groups defined by pre- versus post-pandemic enrollment, and with versus without an ACP discussion or an advance care directive. Some percentages do not add to 100% due to missing data, which we considered to be a separate category.

## Results

### Study sample

The study was launched in early 2020 after receiving IRB approval, but was subsequently put on-hold for a period of time due to the COVID-19 pandemic. Participant recruitment resumed in 2021. A total of 152 patients were initially assessed for eligibility, of which 7 patients had recently passed away. Among the remaining 145 patients, 3 were excluded because they were not receiving care at our institution, and 1 patient was not able to provide informed consent. An additional 41 patients declined to participate (due to lack of interest, not feeling well enough, or privacy concerns), resulting in a sample of 100 patients who consented to participate in the study.

Participants were, on average, 64.5 years of age (SD = 10.0). Fifty-three percent of participants were male, 71% married or living with a partner and 84% self-identified their race as White. Of the 96% with education data, 53% had less than a college degree, 27% had obtained a college degree and 16% had a post-graduate degree. Primary cancer sites included thoracic (29%), gastrointestinal (21%), genitourinary (20%), and head and neck (10%), among others. Participant characteristics are presented in Table 1.

**Table 1.** Participant characteristics (N = 100)

Mean age in years (SD)	64.5 (10.0)
Sex (3 missing)	
Male	53 (53%)
Female	44 (44%)
Race	
Black	11 (11%)
White	84 (84%)
Hispanic or Latino ethnicity	3 (3%)
Marital status (3 missing)	
Not married	26 (26%)
Married	71 (71%)
Educational level (3 missing)	
Less than college	53 (53%)
College degree	27 (27%)
Post-graduate degree	16 (16%)
Disease site (1 missing)	
Thoracic	29 (29%)
Gastrointestinal	21 (21%)
Genitourinary	20 (20%)
Head & neck	10 (10%)
Gynecologic	9 (9%)
Breast	5 (5%)
Hematologic	3 (3%)
Skin	2 (2%)
ECOG status (4 missing)	
0	38 (38%)
1	46 (46%)
2–3	12 (12%)
Advance directive received (1 missing)	
No	59 (59%)
Yes	40 (40%)

### ACP experience

With regard to patients’ experiences, 63% of participants said that they heard about ACP. When participants were asked how much they have thought about ACP, 25% said they thought about ACP a great deal and 48% of patients said they had thought about ACP some, with the remaining participants indicating “not much” (17%), “not at all” (9%), or no response (1%).

On a scale of importance, 34% of participants said ACP was very important; 38% said it was important; and 24% believed it was somewhat important. Regarding how comfortable participants were in having an ACP discussion, only 2% said they were “not comfortable at all” and 7% “not too comfortable”; the rest were somewhat comfortable (38%) or very comfortable (51%). Those who had discussions were much more likely to state that they were very comfortable with having an ACP discussion (73.9%) compared with those who had not had discussions (44.2%) p = 0.012.

While 81% stated they thought health care providers should have ACP discussions with their patients, only 23% stated they had previous discussions with a doctor or other healthcare provider. Interestingly, an advance care directive document was filed in 40% of the participants’ EMR. Of further interest, more had discussions after the start of the pandemic (25/51 = 51%) vs pre-pandemic (13/48 = 27%), p = 0.023. There were no demographic or clinical differences between those participants who did vs. did not have ACP discussions (all p-values > 0.05). Similarly, no differences were observed between those who did vs. did not have an advance care directive filed in the EMR.

Among the 23 participants who reported having an ACP discussion, the following results describe their experiences. Table 2 shows the distribution of responses regarding who the participants talked with about ACP. Of the 23, 17 (74%) reported speaking with their family members about ACP, and 11 (48%) had spoken with their oncologist. Relatively fewer reported having ACP discussions with their family doctor or staff, hospital doctor or staff, a healthcare specialist, or a long-term care facility doctor or staff. Of the 14 participants who had the discussion at our cancer center, 7 (50%) reported being very satisfied, 4 (29%) were satisfied, and 2 (14%) were somewhat satisfied with the discussions they had. The topics discussed with participants are also noted in Table 2.

**Table 2.** Participants who had an ACP discussion (N = 23)

**Who did you talk with about ACP?**	N (%)
Family members	17 (74%)
Oncologist	11 (48%)
Family doctor, nurse, other medical staff	9 (39%)
Hospital doctor, nurse, social worker	8 (35%)
Specialist (cardiologist, pulmonologist, gynecologist) or other doctor	3 (13%)
Long-term care facility doctor, nurse or staff	1 (4%)
Other (lawyer, close friend, caregiver)	5 (22%)
**Topics discussed during ACP discussions**	
Asked what treatments you prefer to have or not to have if you were to develop a life-threatening illness	16 (70%)
Asked if you had prior discussions or written documents about ACP	15 (65%)
Spoke with you about your prognosis (life expectancy or predicted course of illness	15 (65%)
Asked what was important to you as you considered health care decisions at this stage of your life (i.e., values, spiritual beliefs, etc.)	14 (61%)
Given the opportunity to express your fears or discuss your concerns	13 (57%)

### Barriers to ACP

Among those participants (n = 77) who responded they had not had an ACP discussion with a doctor or other health care worker, the reasons for not having an ACP discussion are noted in Table 3. The most common reasons reported were the patient’s preference to speak with family members first and not perceiving oneself to be sick enough to need such a discussion yet. Some participants also believed that their spouse or family members would know the participant’s preferences. Ten (13%) of the respondents noted that this topic is uncomfortable and this was a reason why they had not had an ACP discussion. About 9 (12%) reported that they were waiting for their doctor to start the conversation and 9 (12%) indicated that they trusted their healthcare team to make these decisions for them.

**Table 3.** Reasons for not having an ACP discussion (N = 77)

Reasons	N (%)
I prefer to speak to family about my wishes first	36 (47%)
I am not sick enough to need one yet	30 (39%)
My spouse or family member will know what I want	23 (30%)
The topic makes me uncomfortable	10 (13%)
I will trust the healthcare team to make the decision	9 (12%)
I am waiting for the doctor to start the conversation	9 (12%)
If I choose one person to carry out my wishes, it will upset others	2 3%)
I do not have a person who would carry out my wishes	0 (0%)
The person(s) I wanted to speak with did not want to discuss the topic	0 (0%)
I do not have anyone to discuss the topic with	0 (0%)

Although 77 participants reported that they had not had an ACP discussion, 35 (46%) stated they would want to have one and 46 (60%) would be very comfortable having it with their doctor or other health care provider. These 77 participants would also be very comfortable having conversations with others, including spouse/partner (49, 64%), children (33, 43%), minister/priest/other religious or spiritual advisor (17, 22%), mental health counselor/therapist or other family members (both 15, 20%). Fourteen (18%) reported being very comfortable with having ACP discussion with close friends.

## Discussion

Despite 63% of participants having heard of ACP, and over three quarters saying that health care providers should have ACP discussions, less than 25% reported actually having had ACP discussions with a doctor or other healthcare provider. In addition, nearly half of the participants reported thinking about ACP a great deal, with a third reporting that ACP was very important, and over half reporting they were very comfortable having an ACP discussion. Among the majority of participants who had no prior ACP discussion, the most commonly reported reasons were preferring to speak with family members first, not feeling sick enough to need an ACP discussion yet, and believing that one’s spouse/family members know what the patient’s preferences are. Interestingly, while 23% of participants reported having an ACP discussion, ACP documents were discovered in 40% of the participants’ EMR. Of the 40% who had an advance directive received, only 8 (20%) reported having a discussion with a doctor or other health care provider. It is not clear if participants who had ACP documents understood that their documented wishes were a form of ACP, even though the definition of ACP was shared at the beginning of the questionnaire.

Several of our findings are consistent with those cited in prior literature. For instance, in the Kaiser Health Tracking Poll (Davis et al. 2023b), while about 9 in 10 respondents said doctors should have these discussions with their patients, few actually had these discussions with a doctor or other health care provider. In addition, over 50% reported that they would feel very comfortable having ACP discussions, similar to the 51% reported in our study. In a statewide survey on end-of-life issues conducted by Lake Research Partners for the California Health Care Foundation in 2011, 38% of the participants reported they had heard of advance directives, compared to 63% in our study (Wright et al. 2008). While 47% stated they would like to speak with their doctor about end-of-life wishes if they were seriously ill, only 7% said their doctor talked with them about their wishes. In the present study, some patients reported that the reason why they had not had an ACP discussion was because they were waiting for their doctor to raise this topic. Thus, the suboptimal rates at which ACP discussions are occurring may be due in part to perceptions about whose responsibility it is to start this conversation.

Patients are interested in ACP, but most are still not having these discussions. In our study nearly half stated they prefer to speak to family about their wishes first, while more than a third felt they were not sick enough to need such a discussion and more than a quarter believed their spouse or family member will know their wishes. Some patients rely on their doctor to be more active in this process, trusting them to make the decision or waiting for them to initiate this discussion. Not having anyone to discuss the topic with or to carry out wishes was not perceived to be a barrier to having such discussions. It was also not the case that the person they wished to speak with did not want to discuss the topic. Our findings are similar to those reported by Clark and colleagues (Clark et al. 2018), in which more than one-third of the participants had never had an ACP conversation with a healthcare provider and did not want to do so. Consistent with our findings, the most commonly endorsed reasons in the Clark et al. study were because respondents preferred to speak first to family members or because they were not sick enough and did not feel it was necessary. Many also trusted their health-care team to make decisions when needed (Clark et al. 2018).

Among those participants in our study who reported having had ACP discussions, all had spoken with their family members, and many had spoken with their oncologist. Topics most often discussed included treatment preferences, prognosis, and prior discussions or written documents. These results were similar to those documented in Clark et al.’s study, where over half of all participants reported having a conversation with someone other than a healthcare provider (Clark et al. 2018). Overall, our findings are similar to previously reported findings in the literature (Clark et al. 2018; Davis et al. 2023b; Wright et al. 2008) and identify potential opportunities and strategies for improvement. Learning that ACP was ranked as very important for the majority of our participants should attenuate fears and misperceptions that patients do not want to have such discussions. In addition, the finding that some participants reported that they were waiting for their doctor to start the conversation can be used as an opportunity for improvement in the patient experience. The discrepancy noted between the 40% of participants who had an ACP document filed in the EMR versus 23% of participants reporting that they had an ACP conversation supports the need for improving patient education about ACP planning. Finally, additional qualitative studies are needed to understand the concerns among those respondents who reported being uncomfortable with having an ACP discussion. In particular, the roles of prognostic awareness and prognostic concordance, which relate to the accuracy of patients’ understanding of their prognosis and how that understanding affects their care, will be important to explore as potential barriers to or perceived consequences of ACP discussions. Ultimately, these data can be utilized to develop multi-pronged strategies for enhancing the rate of ACP conversations.

The present study had several limitations. The study recruitment phase was prolonged due to the COVID-19 pandemic. We believe that this could have also negatively impacted the number of participants who enrolled in the study. It is possible that patients who were very uncomfortable with the topic of ACP were less likely to consent to participate; if so, this could have resulted in a sample with a higher proportion of patients who were comfortable with ACP, had given it some thought, and felt that it was an important topic. However, our findings are comparable to those reported in prior studies and replicate earlier results demonstrating that most patients are not having ACP discussions with their oncologist or healthcare team.

### Conclusion

Many patients have heard about ACP and believe that it is important to have these discussions. Many are also comfortable with – and think their health care providers should have – these conversations. Although most patients are engaging in these conversations with family members, conversations are not occurring often enough between clinicians and patients with advanced-stage cancer. Interventions that can effectively improve the rate of conversations between patients and health care providers are needed, perhaps even prior to patients progressing to advance-stage disease. Disseminating these findings among health care providers is a key first step, followed by designing a more robust ACP patient education program. Engaging family members when having conversations with patients may also be beneficial when raising this topic. Ultimately, the identification of strategies for enhancing the ACP process in the oncology context will promote goal-concordant care and improve patient outcomes.
